# Toward a fully wireless endovascular neural interface: Evaluating power transfer efficacy

**DOI:** 10.1371/journal.pone.0351138

**Published:** 2026-06-15

**Authors:** Yi-De Tai, Joel Villalobos, Nima Wickramasinghe, Bryce Widdicombe, Ranjith R. Unnithan, David B. Grayden, Sam E. John

**Affiliations:** 1 Department of Biomedical Engineering, The University of Melbourne, Melbourne, Parkville, Australia; 2 Department of Electrical and Electronic Engineering, The University of Melbourne, Melbourne, Parkville, Australia; 3 Graeme Clark Institute for Biomedical Engineering, The University of Melbourne, Melbourne, Parkville, Australia; 4 Florey Institute of Neuroscience and Mental Health, Melbourne, Parkville, Australia; KIST: Korea Institute of Science and Technology, GERMANY

## Abstract

**Background:**

Endovascular neural interfaces (ENIs) offer a minimally invasive approach for neural stimulation and recording without the need for open brain surgery. However, current generation devices have long transvascular wires from the implant site to the chest. Eliminating these wires will unlock clinical usability, including lowering infection risk from transvascular wires, reducing the risk of thrombosis from altered hemodynamics, and improving mechanical reliability. However, removing these transvascular wires would require efficient power transfer across the skull and tissue while meeting specific absorption rate (SAR) limits, which is a significant challenge in the field.

**Objective:**

This work designed and evaluated endovascular receiver (Rx) and transmitter (Tx) coils within endovascular geometric and biological constraints to maximize wireless power transfer.

**Methods:**

This study evaluated the optimal operating frequencies, quantified coupling, coil quality factors, power transfer efficiency, and SAR using computational modeling, benchtop, and in-vivo testing. The study also assessed the tolerance to coil misalignment and load mismatch. We evaluated each case with and without ferrites with measurements in air, sheep tissue, and in vivo in sheep.

**Results:**

The results showed that inductive power transfer delivered power to endovascular geometry devices at clinically relevant depths. The maximum power transfer efficiency (PTE) reached 11% at 15 mm and 2% at 30 mm, with up to 72 mW delivered at 30 mm under SAR safety limits. The rectangular planar coil pair performed best at ≤15 mm, whereas the ferrite-core flux-pipe Tx with a helical Rx outperformed beyond ~20 mm and was more tolerant to misalignment.

**Conclusion:**

This study demonstrated the feasibility of wirelessly powering multichannel ENIs using coils that can be placed inside a blood vessel and powered inductively. Making an endovascular neural interface fully wireless has the potential to transform the technology by improving both safety and reliability.

## Introduction

**Fig 1:**
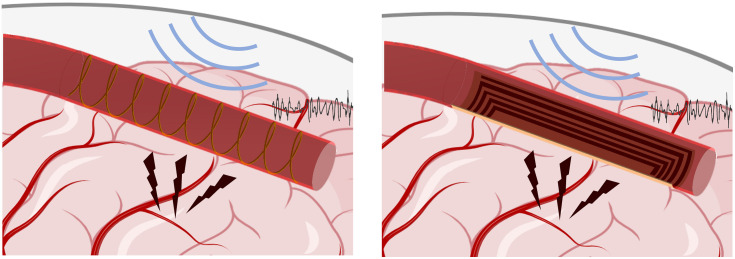
Illustration of wireless ENIs for neural stimulation and recording with helical (left) or planar (right) coils.

Implantable devices, such as deep brain stimulation (DBS) [1], microelectrode arrays (MEAs) [2] and cochlear implants [3] have been widely used to treat the symptoms of neurological disorders and restore lost function. These methods require invasive implantation into the brain or body to stimulate or record neural activity, which is associated with surgical risks and inflammatory responses [4]. The endovascular neural interface (ENI) has emerged as a promising alternative minimally invasive technology for neural stimulation and recording. The ENI uses electrodes placed inside a blood vessels to record neural signals [4,5] and stimulate neural tissue [6–9] in the central or peripheral nervous systems, avoiding more invasive surgery such as open-brain or burr hole surgery. However, existing endovascular neural interfaces (ENIs) rely on long lead wires (50 cm) to connect the electrode array to the implanted wireless device in the chest. This chest-implanted device wirelessly communicated to the external device for power and data transfer [10]. The intravascular wires can cause foreign body responses and lead to altered hemodynamics [11,12]. The physical stress from curved vascular paths and patient movement can lead to wire wear and breakage over time, eventually causing device failure [4]. Furthermore, the ENI must exit the blood vessel to connect to the chest-implanted device, creating a potential route for transvascular infections [13,14]. Ideally, ENIs would operate wirelessly, eliminating the need for wired power and data transfer (Fig 1).

Eliminating intravascular wires will enable a fully implanted device, which can significantly improve its clinical usability. It would reduce infection risk from biofilms that typically form on catheters and remove the transvascular path for bacteria to enter the bloodstream. It would also reduce the risk of stenosis or thrombosis caused by endovascular wires along the 50 cm vessel length, which can irritate the endothelium [13,14]. These wires may also cross venous junctions and valves, significantly altering blood flow [11,12]. Finally, it would improve mechanical reliability by eliminating the dominant failure mode of lead fatigue, reported in early animal studies and potentially present in human studies with multi-electrode decoding [4]. Clinically, this translates to a reduced risk from endovascular implantation. However, achieving stable and sufficient power transfer to endovascular devices presents several challenges.

Few attempts have been made to realize a fully wireless endovascular neural interface. Magnetoelectric thin film has been proposed as a wireless endovascular receiver [15], but its 2 mm thickness would obstruct the blood flow if fully implanted inside the vessel. Ultrasound has also been explored as the wireless link [16]. However, since the skull attenuates a significant proportion of ultrasound, the reported maximum power transfer was limited to 10 mW [17]. Capacitive links has also been demonstrated, achieving up to 2.6% PTE, but the receiver designs required the implantation of two interconnected stents, adding significant surgical complexity [18,19]. Given these limitations associated with these wireless modalities, most reported wireless endovascular devices have relied on inductive coupling (Table 1), with either planar or helical topologies for their implementation and with varying implant sizes from 3 mm diameter to 16 mm diameter. These devices were primarily designed for temporary hemodynamic monitoring [25–31] or hyperthermia [32] applications, rather than long-term neural interfaces, they lack a wearable and compact transmitter (Tx) coil design.

**Table 1:** Wireless endovascular devices reported in the literature.

Reference	Frequency	Shape	Q	Coil Size	Turns	Use	PTE
[24]	182-211 MHz	Planar	N/A	3.6 mm × 7 mm	6	Pressure sensor	N/A
[25]	173-183 MHz	Planar	10	3 mm × 3 mm	15	Pressure sensor	N/A
[26]	103-107 MHz	Planar	N/A	6 mm × 6 mm	30	Pressure sensor	N/A
[27]	5.2 MHz	Planar	N/A	16 mm × 16 mm	15	Pressure sensor	N/A
[28]	4 MHz	Planar	N/A	6 mm × 6 mm	30	Flow sensor	N/A
[29]	70-110 MHz	Helical	N/A	(2.5 mm)^2^π × 20 mm	27	Pressure, pulse, flow sensors	N/A
[30]	50-80 MHz	Helical	N/A	(2.5 mm)^2^π × 20 mm	16	Pressure sensor	N/A
[31]	70 MHz	Helical	11.4	(3 mm)^2^π × 20 mm	16	Hyperthermia	6.8% at 15 mm (measurement with bulky external coil)
[20]	13.56 MHz	Planar	N/A	2.8 mm × 15 mm	5	Pacemaker	N/A
[21]	1 MHz	Planar	N/A	8 mm × 15 mm	6	Pacemaker	3.5% at 20 mm (simulation without tissue)
This work	13.56 MHz	Planar	20	3 mm × 30 mm	26	General-purpose	11.2% at 15 mm (simulation + measurement)
This work	6.78 MHz	Helical	17	(1.5 mm)^2^π × 30 mm	30	General-purpose	8.3% at 15 mm (simulation + measurement)
This work	13.56 MHz	Helical	25	3 mm × 30 mm	30	General-purpose	3.0% at 15 mm (simulation + measurement)

**Fig 2:**
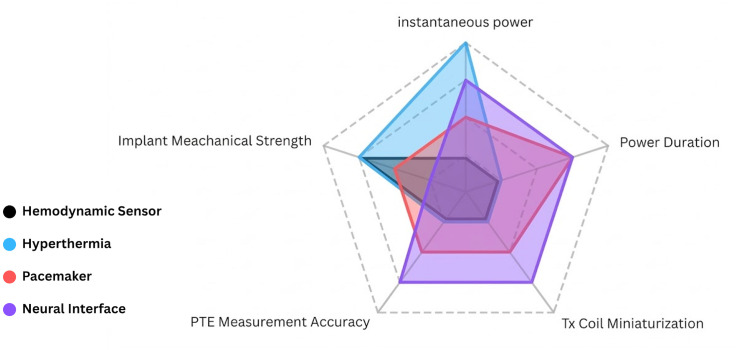
The design requirements for different types of endovascular devices.

Recent work on leadless pacemakers has demonstrated stimulation of cardiac tissue without transvascular wire connections [20,21]. For continuous treatment, stable wireless power links were proposed and power transfer efficiency (PTE) was calculated [21]. However, the reported values were based solely on theoretical simulations, without experimental validation. Critically, the simulations did not account for the parasitic capacitance and conductance of biological tissue, which are known to significantly reduce the quality factor of coils up to 50% and consequently degrade the overall PTE [22]. For neural interface applications, continuous monitoring and stimulation would be required, necessitating a stable link with a compact wearable Tx. Neural recording typically consumes only several μW per channel, whereas neural stimulation, such as deep brain stimulation (DBS), can demand up to 10 mW per channel [23], which is significantly higher than the power consumption demonstrated in leadless pacemakers. In such cases, wireless power transfer raises safety concerns, as exposure to high electromagnetic fields may cause tissue heating. This imposes limits on the maximum power delivered to the load (PDL). However, both safety constraints and PDL have not been thoroughly investigated for wireless endovascular devices. Fig 2 compares the design requirements for the aforementioned endovascular devices.

The strength of wireless links through magnetic inductions is invariant across underlying tissue [32], only dependent on the implant depth. In Table 2, potential vessels for ENI implantations are listed. They are generally 10–30 mm deep from the skin surface. To operate reliably across this depth range, we designed multiple wireless Tx and Rx coil pairs optimized for ENIs. Combining both computational and experimental data, our work extends previous studies on wireless power transfer to endovascular implants by developing compact wearable Tx coils suitable for chronic applications, systematically characterizing power-transfer efficiency (PTE) and specific absorption rate (SAR) in tissue and identifying 6.78 MHz and 13.56 MHz as suitable operating frequencies. In addition, we quantified achievable power delivery under SAR safety limits, demonstrating up to 72 mW at 30 mm, and evaluated misalignment tolerance to ensure robust powering in realistic in-vivo conditions. These advances provide practical design guidance for wireless powering of multichannel ENIs.

**Table 2:** Potential vessels for ENI implantations.

Potential Vessel	Target brain region/nerve	Estimated Vessel Depth	Reference
Superior sagittal sinus (anterior)	Motor Cortex	10-20 mm	[4,6,33,34]
Superior sagittal sinus (posterior)	Visual Cortex	15-25 mm	[33–35]
Transverse sinus	Cerebellum	20-30 mm	[33,34]
Femoral artery	Femoral nerve	15-30 mm	[15,36]
Internal jugular vein	Vagus nerve	10-25 mm	[37,38]
Upper-limb vessels	Median/radial/ulnar nerve	10-20 mm	[39,40]
Lower-limb vessels	Peroneal nerve	10-30 mm	[41]

## Methods

### Finite Element Analysis (FEA)

COMSOL Multiphysics 6.0 (COMSOL, Inc., USA) was used for modeling magnetic fields of Tx and Rx coils. In COMSOL’s Magnetic Field Physics module, the outer boundaries of the computational domain were set to Magnetic Insulation (n·A = 0), constraining normal magnetic flux to zero as an approximation of free-space conditions. Helical coils were modelled with the built-in homogenized multiturn circular coil. Planar coils were simplified to Edge Currents ($I_{\text{Tx}}$, $I_{\text{Rx}}$) to reduce computational load, where coils of unconventional shapes such as the bent endovascular coils could be modeled with low complexity. Coil inductances ($L_{\text{Tx}}$, $L_{\text{Rx}}$) were calculated from the Global Evaluation of the total magnetic energy ($U_{\text{m}}$),


$$\begin{array}{c} \begin{array}{c} U_{\text{m}}=\frac{LI^{\text{2}}}{\text{2}}.\; \end{array} \end{array}$$ (1)

The induced voltages ($V_{\text{Rx}})$ of the coils were computed from the Line Integral of the electric field, and the mutual inductance ($L_{m}$) was thus calculated from


$$\begin{array}{c} \begin{array}{c} L_{m}=\frac{V_{\text{Rx}}}{\omega I_{\text{Tx}}}. \end{array} \end{array}$$ (2)

For helical endovascular coils, a built-in homogenized multiturn circular coil model was used, where the computation of inductance and induced voltage was built in. With simulations of $L_{\text{Tx}}$, $L_{\text{Rx}}$, and $L_{m}$, *k* could be calculated from


$$\begin{array}{c} \begin{array}{c} k=\frac{L_{m}}{\sqrt{L_{\text{Tx}}L_{\text{Rx}}}}.\; \end{array} \end{array}$$ (3)

However, the resistance of the coils and the parasitic capacitance due to tissue could not be modeled using this method. Hence, $Q_{\text{Tx}}$ and $Q_{\text{Rx}}$ values were determined experimentally.

Sim4Life 6.0 (ZMT Zürich MedTech AG, Switzerland) was used for SAR simulation due to its built-in SAR evaluator (Peak Spatial-Average SAR[IEEE/IEC62704–1] (1g)). Tx coils were constructed using the Coil in Templates. The magnetic field generations were simulated in the Magneto Static (Biot-Savart) model. The generated field was then used as the Source in the Magneto Quasi-Static model in Sim4Life, where the resulting electric fields in tissue layers were computed. In Analysis, the SAR Statistics in Dosimetry was used. The frequency-dependent tissue conductivity (σ) was extracted from [42].

### Coil fabrication

Planar Rx coils were printed on flexible polyimide substrates via a commercial manufacturer (PCBWay, China). Helical Rx coils were manually wound with copper wires of 0.1 mm diameter. All Tx coils were manually wound with copper wires of 0.5 mm diameter. The ferrites used were model 364104 from Wurth Elektronik, Germany.

### Coil measurement

To measure the voltage induced at the Rx, a class-E amplifier was built to provide high-current AC to drive the Tx coils ($I_{\text{Tx}}$). The switching transistor in the amplifier was a Gallium Nitride (GaN) transistor (TP65H150G4PS, Transphorm, USA), whose gate was controlled by square wave at the operational frequency (ω), generated by a signal generator. The DC source was supplied by a DC power supply. $I_{\text{Tx}}$ was measured by a current probe (CT6710, Hioki, Japan) connecting to an oscilloscope (TBS 1000C, Tektronix, USA). The Rx coil was in a left open-circuit configuration, and the induced voltage ($V_{\text{Rx}}$) was observed on the oscilloscope through a voltage probe. The Rx coils were inserted into plastic tubes of 3-mm diameter. A ruler and a protractor were used to measure the inter-coil distances and misalignment angles.

$Q_{\text{Tx}}$ and $Q_{\text{Rx}}$ were measured using the NanoVNA (ZeenKo, China). To mimic the implantation environment, lamb tissue was placed beneath the Tx and surrounding the Rx. The Rx coils were inserted into plastic tubes of 3-mm diameter filled with saline.

### Coil implantation

All animal protocols were approved by the Animal Ethics Committee of the Florey Institute of Neuroscience and Mental Health, Melbourne, Australia (approval number: 22–010-UM). Farm-sourced sheep underwent surgery under terminal anesthesia with inhaled isoflurane in air (2–4%). For measurement of the inductive link in vivo, sub-scalp space (around 10 mm deep) and subdural space (around 20 mm deep) were reached through a craniotomy and for inserting the Rx coils. Tx coils were placed on the top of scalp. The same instruments and procedures were used for measuring voltage induction. At the completion of the experiment, the animals were euthanized under anesthesia with an overdose of sodium pentobarbitone (100 mg/kg, i.v.).

## Results

### Design of coil geometry

Endovascular Rx coils in previous work (Table 1) have two distinct shapes: Planar and helical (Fig 1). Planar coils are printed on flexible substrates and can wrap around the blood vessel wall, so their widths are constrained by the vessel diameter. Planar coils in previous work are mostly square-shaped and connected to stents as embedded sensors. In the context of ENIs, the stent itself is not required to maintain patency of the vessel or mechanical support. Theoretically, a rectangular coil, of the same size of a stent, could be used as the primary structure, thereby increasing the effective coil size to capture magnetic flux and improve the receiver efficiency. Helical coils naturally resemble the tubular shape of a stent. A helical coil with the same diameter as the vessel can be formed by reshaping the stent into a helix structure. In this work, we considered both types of endovascular coils. We set each coil to a length of 30 mm and configured them for 3 mm diameter vessels, consistent with previously reported dimensions ^4,6^.

**Fig 3:**
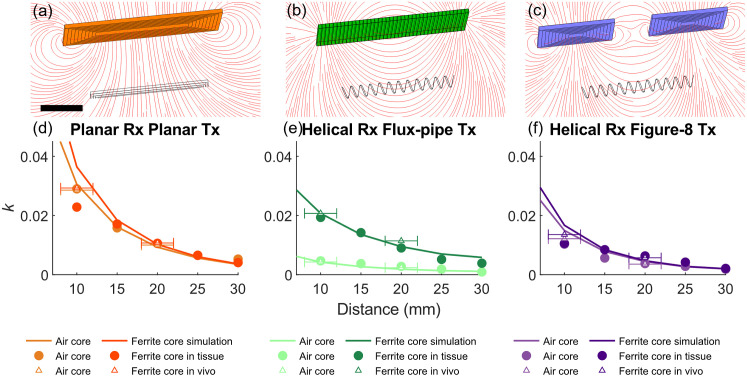
Wireless link simulation and measurement at 10 MHz. COMSOL simulation of magnetic field induced by the designed (a) planar Tx designed for planar Rx, (b) flux-pipe Tx designed for helical Rx, and (c) figure-8 Tx for helical Rx. Red lines indicating the magnetic field lines and colored solids represent ferrites. (d-f) *k* versus inter-coil distance calculated from simulation (lines), tissue measurements (dots), and in-vivo measurements (triangles). Error bars indicate the uncertainties of implant distances. Scale bar = 10 mm.

Wearable Tx coils attached to the skin were designed to transmit power to endovascular Rx planar or helical coils. To maximize the link strength, the magnetic field generated by both coils should overlap. Since blood vessels are typically tangential to the body surface, the planar Rx placed against the vessel wall produces magnetic field normal to the body surface. Therefore, its corresponding Tx should also be planar and produce magnetic field perpendicular to the body surface. Circular or square coils are predominantly used for planar Tx coils [22,43,44]; we modified them to a rectangular shape to accommodate the rectangular Rx (Fig 3a). For helical Rx, however, the produced magnetic field is tangential to the body surface. To align the Tx magnetic field, flux-pipe coils, adapted from electric vehicle charging [45], can be utilized. They consist of wires wound around a long and thin core (Fig 3b). Another solution employs two planar coils with opposite current directions, mimicking the planar figure-8 coil used in transcranial magnetic stimulation to generate tangential magnetic fields in the brain [46] (Fig 3c). For each of the three Tx shapes, incorporation of ferrites was considered. Ferrites are magnetic materials, which are often used as a core base on which the coil is wound to enhance and concentrate the magnetic fields [45]. The final form of the Tx coils was configured to 10 mm × 40 mm × 1 mm in balance of compactness and strength.

To quantify the link strength of the three Tx-Rx coil pairs, the coupling coefficient *k* between Tx and Rx was simulated in COMSOL and measured in tissue and in vivo (Fig 2d-2f). Among the three coil pairs, the planar pair with ferrite resulted in the highest *k* in simulation, ranging from 0.031 at 10 mm to 0.004 at 30 mm. The flux-pipe-helical pair with an air core exhibited the lowest *k*, ranging from 0.004 at 10 mm to 0.001 at 30 mm. At any distance, while ferrite cores only increased *k* in the planar pairs and figure-8-helical pair for no more than 30%, ferrite cores increased *k* in the flux-pipe helical pair for over 450%. With a ferrite core, the flux-pipe-helical pair maintained strong coupling with a slow decay (from 0.021 at 10 mm to 0.006 at 30 mm), the results from both tissue and in-vivo measurements aligned with the simulation values.

### Identification of operational frequency

In previous work on wireless endovascular devices, the operational frequencies ranged from 1 MHz to 200 MHz. These various values were either preselected before circuits were designed or simply determined by the sensor capacitor values. We aimed to identify the operational frequency that yielded the highest PTE based on the quality factors (*Q*) of the Tx and Rx coil.

**Fig 4:**
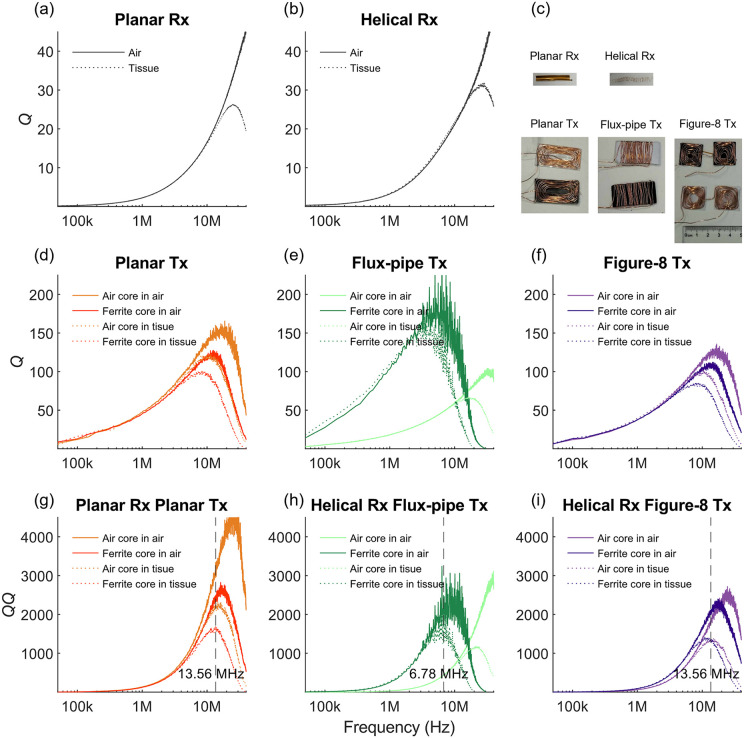
Coil measurements across frequency sweeps. ${𝐐}_{\text{Rx}}$ of the (a) planar Rx coil and the (b) helical Rx coil, measured in air (solid lines) and in tissue (dashed lines). **(c)** Rx and Tx coil prototypes. ${𝐐}_{\text{Tx}}$ of the **(d)** planar Tx coil, the (e) flux-pipe Tx coil **(e)**, and the **(f)** figure-8 coil, measured in air (solid lines) and in tissue (dashed lines). ${𝐐}_{\text{Rx}}Q_{\text{Tx}}$ of the (g) planar-planar pair, the (h) helical-flux-pipe pair **(h)**, and the **(i)** helical-figure-8 pair, measured in air (solid lines) and in tissue (dashed lines).

Fig 4 shows Q from 50 kHz to 40 MHz, measured in air and in tissue. $Q_{\text{Rx}}$ for both planar and helical coils increased with frequency in air, but when tissue was present, the peak was at 26 MHz and 27 MHz, respectively (Fig 4a, 4b, S1 Fig). For the planar Tx (Fig 4d), the peak $Q_{\text{Tx}}$ was 153 at 17 MHz in air and 120 at 10 MHz in tissue. The addition of a ferrite base reduced peak $Q_{\text{Tx}}$ to 124 at 13 MHz in air and 100 at 9 MHz in tissue. For the flux-pipe Tx (Fig 4e), the peak $Q_{\text{Tx}}$ was 104 at 32 MHz in air and 65 at 17 MHz in tissue. The addition of a ferrite core significantly boosted $Q_{\text{Tx}}$, with reductions of the peak frequency, to 182 at 6 MHz in air and 153 at 4 MHz in tissue. The trends of the figure-8 Tx (Fig 4f) were similar to the planar Tx, but their magnitudes were slightly lower. The peak $Q_{\text{Tx}}$ was 126 at 17 MHz in air and 109 at 10 MHz in tissue, and 124 at 13 MHz in air and 100 at 9 MHz in tissue with a ferrite base. The observed reduction of Q with the inclusion of ferrite can be attributed to a disproportionate increase in resistance relative to inductance. Although the ferrite increased the coil inductance by approximately 30% (S2a, S2c Fig), it introduced a larger increase in total resistance (S2b, S2d Fig) and this increase dominates over the inductance enhancement, resulting in a net reduction in $Q_{\text{Tx}}$. This additional resistance is likely due to the eddy current or hysteresis loss present in the ferrites at high frequencies [45], as well as the imaginary permeability of the ferrite, will results in internal magnetic loss, according to the ferrite material datasheet [47].

PTE is contributed to by the product of $Q_{\text{Rx}}Q_{\text{Tx}}$ (Fig 4g-4i). For the planar-planar pair in tissue, $Q_{\text{Rx}}Q_{\text{Tx}}$ was 2200 at 15 MHz without ferrite and 1600 at 13 MHz with ferrite. For the helical-flux-pipe pair in tissue, $Q_{\text{Rx}}Q_{\text{Tx}}$ was 1100 at 22 MHz without ferrite and 1700 at 6 MHz with ferrite. For the helical-figure-8 pair in tissue, $Q_{\text{Tx}}Q_{\text{Rx}}$ was 2200 at 15 MHz without ferrite and 1600 at 13 MHz with ferrite. Notably, these peak frequencies aligned closely with the frequency selected in the Industrial, Scientific, and Medical (ISM) bands [48]: 6.78 MHz and 13.56 MHz. Accordingly, we chose 13.56 MHz as the operational frequency for the planar-planar pair and the helical-figure-8 pair and 6.78 MHz for the helical-flux-pipe pair. The two frequencies not only ensure the optimal power link but also provide good compatibility for data link.

### Derivation of power efficiency and maximum power

The equation for the theoretical maximum PTE ($\eta_{\text{max}}$) is [49]:


$$\begin{array}{c} \begin{array}{c} \eta_{\text{max}}=\frac{k^{\text{2}}Q_{\text{Tx}}Q_{\text{Rx}}}{{\left(\sqrt{\text{1}+k^{\text{2}}}+\sqrt{\text{1}+k^{\text{2}}Q_{\text{Tx}}Q_{\text{Rx}}}\right)}^{\text{2}}}.\; \end{array} \end{array}$$ (4)

Using the simulated and measured *k* across distances and the measured Q in tissue at the selected operational frequency, $\eta_{\text{max}}$ was derived. For the helical-flux-pipe pair, the addition of a ferrite core in Tx greatly increased both *k* and $Q_{\text{Tx}}$, so it was retained in the final Tx design. For the planar-planar pair and the helical-figure-8 pair, however, the reduction in $Q_{\text{Tx}}$ caused by ferrite cores outweighed their contribution to higher *k.* Therefore, the ferrites were removed in their final designs.

**Fig 5:**
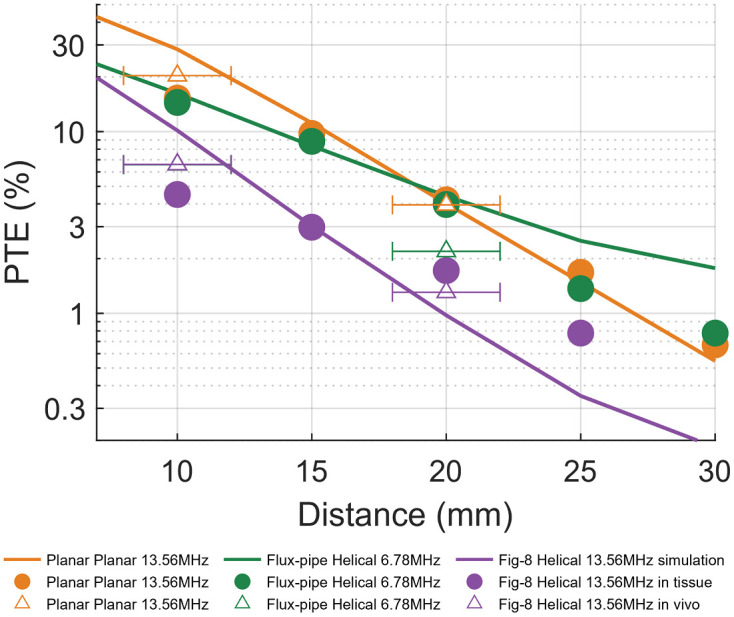
Optimal PTE from simulation (lines), tissue measurements (dots), and in-vivo measurements (triangles), for the three coil pairs versus inter-coil distance. Error bars indicate the uncertainties of implant distances. Copper wires and ferrites were selected for all three Tx coil shapes. Operational frequencies were set at 13.56 MHz for the planar-planar pair and helical-figure-8 pair, and at 6.78 MHz for the helical-flux-pipe pair.

Fig 5 shows the $\eta_{\text{max}}$ of the three pairs at their operational frequencies. The planar-planar pair reached the highest PTE at shorter distances, achieving 28% at 10 mm and 11% at 15 mm with simulated *k*. Meanwhile, the helical-flux-pipe pair exhibited superior performance at longer distances, achieving 4% at 20 mm, 3% at 25 mm, and 2% at 30 mm with simulated *k*, due to its slower decay of *k* with distance. The helical-figure-8 pair achieved the lowest $\eta_{\text{max}}$ among the three pairs at any distance, ranging from 10% at 10 mm to only 0.2% at 30 mm. The measured values for the helical-flux-pipe pair were lower than the simulated values, likely because the ferrite’s magnetic permeability might decrease at high frequencies compared to its original specifications. In contrast, the measured values for the helical-figure-8 pair were lower than the simulated values, possibly due to the lack of complete isolation between the input port for Tx and the output port for Rx in the experimental setup, introducing in artifacts when the measured values were low.

**Fig 6:**
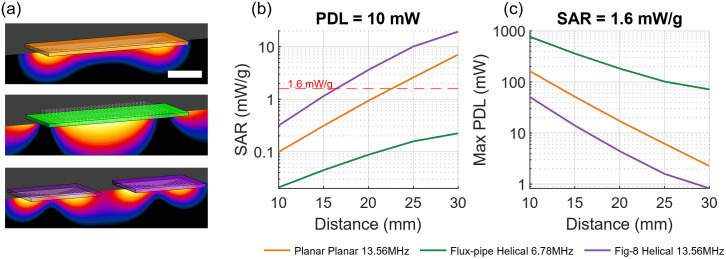
SAR simulations for the three coil pairs at each’s operational frequency. **(a)** The normalized SAR distribution in Sim4Life simulation. **(b)** SAR versus inter-coil distance for 10 mW PDL. **(c)** Maximum PDL versus inter-coil distance under 1.6 mW/g SAR. Scale bar = 10 mm. All conditions were the same except for the operating frequencies were chosen for the highest efficiency for each coil pair.

SAR was simulated using Sim4Life built-in SAR evaluator (Fig 6a). Since the power transmitted by Tx is much higher than the power reflected by Rx, SAR is primarily driven by Tx. We set the target PDL to 10 mW, so the required power transmitted by Tx would be $\text{10 mW}/\eta_{\text{max}}$, for $\eta_{\text{max}}$ of the three pairs across distances. The corresponding current was passed through Tx, to simulate SAR for 10 mW PDL (Fig 6b). For the helical-flux-pipe pair with the lowest PTE, the SAR exceeded the 1.6 mW/g threshold from Federal Communications Commission (FCC) Standard [50] at distances beyond 17 mm. The planar-planar pair exceeded the limitation at distances beyond 23 mm. SAR is proportional to the square of frequency in the inductive near-field. Operating at a lower frequency, the helical-flux-pipe pair generated the lowest SAR, with the value lower than 0.3 mW/g within the 30-mm distance range. When the SAR level was fixed to 1.6 mW/g (Fig 6c), the maximum PDL was 767 mW at 10 mm to 72 mW at 30 mm for the helical-flux-pipe pair, was 163 mW at 10 mm and 2 mW at 30 mm for the planar-planar pair, and was 51 mW and 10 mm to 0.8 mW at 30 mm for the helical-figure-8 pair.

### Investigation on misalignment and mismatch tolerance

Several conditions can lead to degradation of wireless power transfer. The most common issues include frequency detuning, quality factor degradation, coil misalignment, and load mismatch. The first two were investigated above. Here, we investigate coil misalignment and load mismatch.

**Fig 7:**
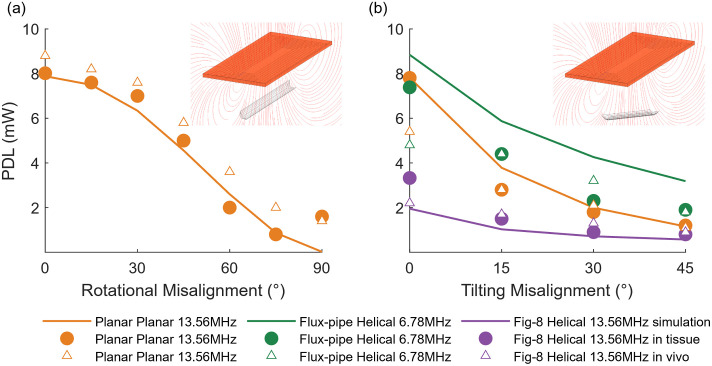
Misalignment investigations for the three coil pairs at each’s operational frequency. **(a)** PDL versus rotational misalignment of the planar Rx coils, and **(b)** PDL versus tilting misalignment for planar and helical Rx coils from simulation (lines), tissue measurements (dots), and in-vivo measurements (triangles). The power source is set to 500 mW and the inter-coil distance is fixed to 20 mm.

Fig 6 showed the impacts of rotational and tilting misalignments, where *k* was simulated and measured. The power source for the Tx coil was set to 500 mW and the distance was set to 20 mm. The PDL was derived using the aforementioned flow. The helical Rx is inherently immune to rotational misalignment due to its cylindrical symmetry. The planar Rx coil, however, experiences reduced magnetic field reception when its surface does not directly face the Tx coil, leading to lower *k* and PDL (Fig 7a). When the rotation was 45°, PDL dropped to 57% of its original. When the rotation was 90°, the two coils became perpendicular and the aligned magnetic fields were minimal, leading to less than 0.3% of their original value at a given distance in simulations. Measured data followed the same trend although higher values were observed in low coupling conditions, likely due to the incomplete isolation discussed earlier.

**Fig 8:**
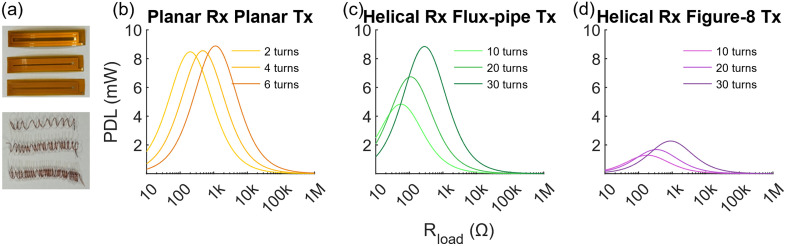
Load matching simulation with different coil turns. **(a)** Rx coil prototypes with 2, 4, and 6 turns for planar Rx and 10, 20, 30 turns for helical Rx. Load resistance versus PDL at 13.56 MHz **(b)** for different turns of planar Rx with planar Tx, **(c)** for different turns of helical Rx with flux-pipe Tx at 6.78 MHz, and **(d)** for different turns of helical Rx with figure-8 Tx at 13.56 MHz. The power source was set to 500 mW and the inter-coil distance was fixed to 20 mm.

Tilting misalignments occur when the targeted vessel for implantation is not perfectly parallel to the body surface, affecting both types of Rx coils (Fig 7b). At 45° misalignment, PDL to the planar Rx dropped to 15% of its original. The helical-flux-pipe pair demonstrated greater tolerance to vessel misalignment, retaining 36% of its original at 45° misalignment. The helical-figure-8 pair had the lowest PDL due to its lowest PTE at the same distance, but also retaining 36% PDL at 45° misalignment. These results suggest the helical Rx is more tolerant of both procedural and anatomical misalignments.

In wireless power transfer, PTE reaches $\eta_{\text{max}}$ only when the load impedance is perfectly matched with the coil impedances. When the load is matched, the PTE value (η) becomes [49]


$$\begin{array}{c} \begin{array}{c} \eta \;=\frac{k^{\text{2}}Q_{\text{Tx}}Q_{\text{Rx}}R_{\text{Tx}}R_{\text{Rx}}R_{\text{Ls}}}{R_{\text{Tx}}{\left(R_{\text{Rx}}+R_{\text{Ls}}\right)}^{\text{2}}+k^{\text{2}}Q_{\text{Tx}}Q_{\text{Rx}}R_{\text{Tx}}R_{\text{Rx}}\left(R_{\text{Rx}}+R_{\text{Ls}}\right)}, \end{array} \end{array}$$ (5)

where $R_{\text{Tx}}$ and $R_{\text{Rx}}$ are the coil resistances of Tx and Rx coils, respectively, and $R_{\text{Ls}}$ is the serial load resistance. For wireless power transfer, a parallel load ($R_{\text{Lp}}$) is more often used for higher load allowance [32], which can be converted as


$$\begin{array}{c} \begin{array}{c} R_{\text{Lp}}≅\frac{\overset{\text{2}}{\underset{\text{Rx}}{Q}}\overset{\text{2}}{\underset{\text{Rx}}{R}}}{R_{\text{Ls}}}. \end{array} \end{array}$$ (6)

Using these equations, PDL of the three pairs, with 500 mW power source and 20 mm distance, were calculated across load resistances (Fig 8b-8d). The original designs, with 6 turns and 30 turns for planar and helical Rx coils, respectively, yielded $\eta_{\text{max}}$ at $R_{\text{Lp}}$ of 1130 Ω, 270 Ω, and 890 Ω, for the planar-planar pair, helical-flux-pipe pair, and helical-figure-8 pair, respectively. The helical-flux-pipe required the lowest $R_{\text{Lp}}$ because its lower operational frequency led to a lower $Q_{\text{Rx}}$. When $R_{\text{Lp}}$ was one order of magnitude higher or lower, PDL for all pairs dropped to 32% ~ 35% of their originals. When the number of turns of the coils was reduced (Fig 7a), the reduced coil resistances shifted the optimal $R_{\text{Lp}}$ to lower values.

## Discussion

This study is the first to calculate the maximum PDL for wireless endovascular devices. Our results demonstrate that up to several hundred mW can be safely delivered to the ENIs implanted up to 30 mm distance while remaining under the SAR limit (Fig 5c). This study establishes the design for fully a wireless endovascular system.

The link efficiency demonstrates feasibility of a fully wireless endovascular neural interface*.* Current endovascular neural interfaces are placed within the superior sagittal sinus in the brain [51,52], which is typically 15 mm deep in human brains. At this depth, our system achieved a PTE of 11%. Other endovascular devices (Table 1) showed 3.5% at 20 mm and 6.5% at 15 mm (Fig 5). Our implantation of the device demonstrates a way to improve this performance above the previously reported values. However, PTE in this study decreased sharply to 2% at the implantation depth of 30 mm, suggesting strong sensitivity of wireless coupling efficiency to implantation depth, anatomical variability, and coil alignment, posing challenges in practical ENI deployment. The operational frequencies in prior studies ranged from 1 MHz to 200 MHz (Table 1). To determine the optimal frequency, we compared $Q_{\text{Tx}}$ and $Q_{\text{Rx}}$ in air and with tissue environments (Fig 4). Our results showed that Q factors started to drop in tissue beyond 20 MHz. Therefore, to achieve the highest PTE, operational frequencies below 20 MHz should be selected.

The findings also highlight the importance of characterizing coil properties in tissue models to accurately mimic implantation conditions. However, it is important to note that the PTE values in this study represented only inductive link efficiency, while the overall PTE in practical use also depends on the efficiency of the power amplifier at the Tx end and the AC-DC conversion efficiency at the Rx end [53], which were not included in this study. The power amplifier used in this work was a class-E amplifier, which is a widely adopted topology for IPT. In theory, class-E amplifiers can achieve nearly 100% efficiency, but only when the inductors, capacitors, and load are precisely matched to their ideal values [54]. Future work will incorporate circuit design and fabrication to achieve the fully wireless ENI.

For neural recording, another feature of importance is data transmission, which could be done using the same inductive link or a separate one [55]. Our designed links were optimized at 6.78 MHz or 13.56 MHz, perfectly matching the frequencies of the ISM bands [48]. This allows simultaneous power and data transmission with a single endovascular Rx coil. The compatibility with ISM bands also facilitates the integration of existing microcontroller design with analog neural recording front ends. However, if the recording signals require higher bandwidth, a separate coil or antenna operating at a higher frequency would be necessary. Furthermore, operation within frequency bands requires coil and circuit tuning to the resonance frequency, which could suffer from detuning effects caused by physiological motion, tissues heterogeneity, and coil deformation following implantation.

This study addressed the upper power limit of 72 mW to wireless endovascular devices based on SAR constraint simulation, serving as a constraint for scalability or high-power applications. Nevertheless, since one channel requires only a few μW for recording and less than 10 mW for stimulation [23], the budget is still sufficient to support multichannel ENI application. Furthermore, when SAR constraints are satisfied, high power dissipated by an implantable device may still lead to adverse tissue heating exceeding 2 ºC. Previous studies have shown that a power dissipation of 40 mW from brain implants can cause a local temperature elevation of 2ºC [56]. Our measurement of coil heating yielded similar results (S3 Fig). For endovascular implantations, heating issues are expected to be mitigated by the cooling effect of blood flow. Since the computational model may not fully capture the complex thermal and electromagnetic interaction in vivo, future studies should attempt to measure actual tissue heating using in-vivo studies.

This study is the first to demonstrate feasibility of inductive power transfer to a device with an endovascular geometry at realistic depths and the impacts of misalignment. Comparing the two candidates of the endovascular Rx coils, the planar coils show the better power transfer when the inter-coil distance is shorter than 15 mm. Beyond the distance, the helical coils perform better. Coil misalignments include lateral or angular misalignments, resulting in lower *k* and thus lower PTE. Lateral misalignments can be mitigated by repositioning the external Tx coil to align it with the implanted Rx coil and achieve highest link strength, so they were excluded from this study. Angular misalignments, however, are more difficult to control, particularly for endovascular devices where misalignments may be caused by procedural (rotation of the Rx) or anatomical (tilting of the blood vessel) factors. Our results suggest the helical Rx is more tolerant to both procedural and anatomical misalignments than the planar Rx. Even when the geometry is aligned, load misalignments could result in lower power delivered to the implant circuit. All these misalignments could reduce the PTE from its initial estimation in dynamic in-vivo environments. Moreover, a single-receiver, single-load configuration was employed in this scheme. In scenarios involving multiple receivers for simultaneously operating multiple devices or multiple channels, mutual coupling and inter-channel interference should be considered.

Recent work has demonstrated the use of an endovascular neural interface in submillimeter-scale vasculature to achieve single-cell resolutions [57]. However, miniaturizing Rx coils to accommodate smaller vessels leads to substantial efficiency reductions. In previous investigation, a reduction of Rx coil by 4 times in length decreased PTE more than tenfold at 10 mm implant depth, and more than 60 times at 30 mm implant depth [58]. This depth-dependent degradation indicates that efficiency loss becomes increasingly severe at deeper implantation sites, where smaller vessels are usually located. Furthermore, since these vessels are typically oriented perpendicular, not parallel, to the scalp surface, their endovascular coils require different coil geometric design. Nevertheless, the cerebral vascular networks are interconnected. Therefore, we propose that the coils designed in this study can still be implanted in SSS and wire-connected to smaller electrode arrays in smaller or deeper vessels. This hybrid approach may help preserve wireless link strength while requiring minimal lead length.This study demonstrated proof of concept for wireless powering of endovascular multichannel ENIs and the outlined quantitative performance metrics for power efficiency and power budgets. Future work will focus on the development of a fully implantable system incorporating integrated circuitry and electrodes, as well as the evaluation of long-term device stability and biological tissue safety, with the ultimate goal of translation to clinical applications.

## Conclusions

By combining theoretical analysis, computational modeling, and experimental measurements, we proposed three distinct types of Tx coils: a rectangular planar coil modified from conventional wearable square or circular coils, a flux-pipe coil adapted from electric vehicle, and a figure-8 coil inspired by transcranial magnetic stimulation coils. Notably, while the rectangular coil exhibits the best performance at shorter distances, it was outperformed by the flux-pipe coil beyond 20 mm. The flux-pipe coil also showed better misalignment tolerance. By characterizing Q factors in tissue, we identified 6.78 and 13.56 MHz as suitable operational frequencies with optimal power efficiencies. The results showed the maximum PTE to reach 11% at 15 mm inter-coil distance and 2% at 30 mm and estimated delivery of 72 mW to 30 mm under SAR safety limits. These results demonstrated the feasibility of wireless powering endovascular multichannel ENIs.

## Supporting information

S1 Fig Impedance of all Rx coils (planar and helical with different turns). Blue lines show their magnitudes and orange lines show their phases in air (solid lines) or in tissue (dashed lines). Phases are aligned in air and in tissues, but magnitude increases in tissues beyond 10 MHz.
(TIF)

S2 FigImpedance of the planar Tx coil.(a) Reactances (X=ω×L) in air. (b) Resistances (*R*) in air. (c) Reactances in tissue. (d) Resistances in tissue. Ferrite increased X by approximately 30% (a, c), but it introduced a larger increase in R (b, d), particularly in frequency beyond 10 MHz, matching the frequency range where the imaginary permeability starts to increase according to [47].(TIF)

S3 Fig Measurement of temperature increase of the planar Rx coil vs. its power dissipation. (a) The temperatures were captured by FLIR ETS320 thermal camera. The coil received different amounts of power and its temperature (red mark) was measured at a steady state and compared to surroundings (blue mark). The temperature was recorded from the pixel of the red and blue marks. (b) The coil temperature increase reached 2 °C at 38 mW dissipation, similar to the reported value of 40 mW from [55].
(TIF)

S1 File S-parameter measurements of all coils. (ZIP)

S1 Table Result simulation and measurement data. (XLSX)
