# Supplementary figures and images for: Toward a fully wireless endovascular neural interface: Evaluating power transfer efficacy
Source: PLoS One. 2026 Jun 15;21(6):e0351138. doi: 10.1371/journal.pone.0351138 (PMC13268161; doi:10.1371/journal.pone.0351138)

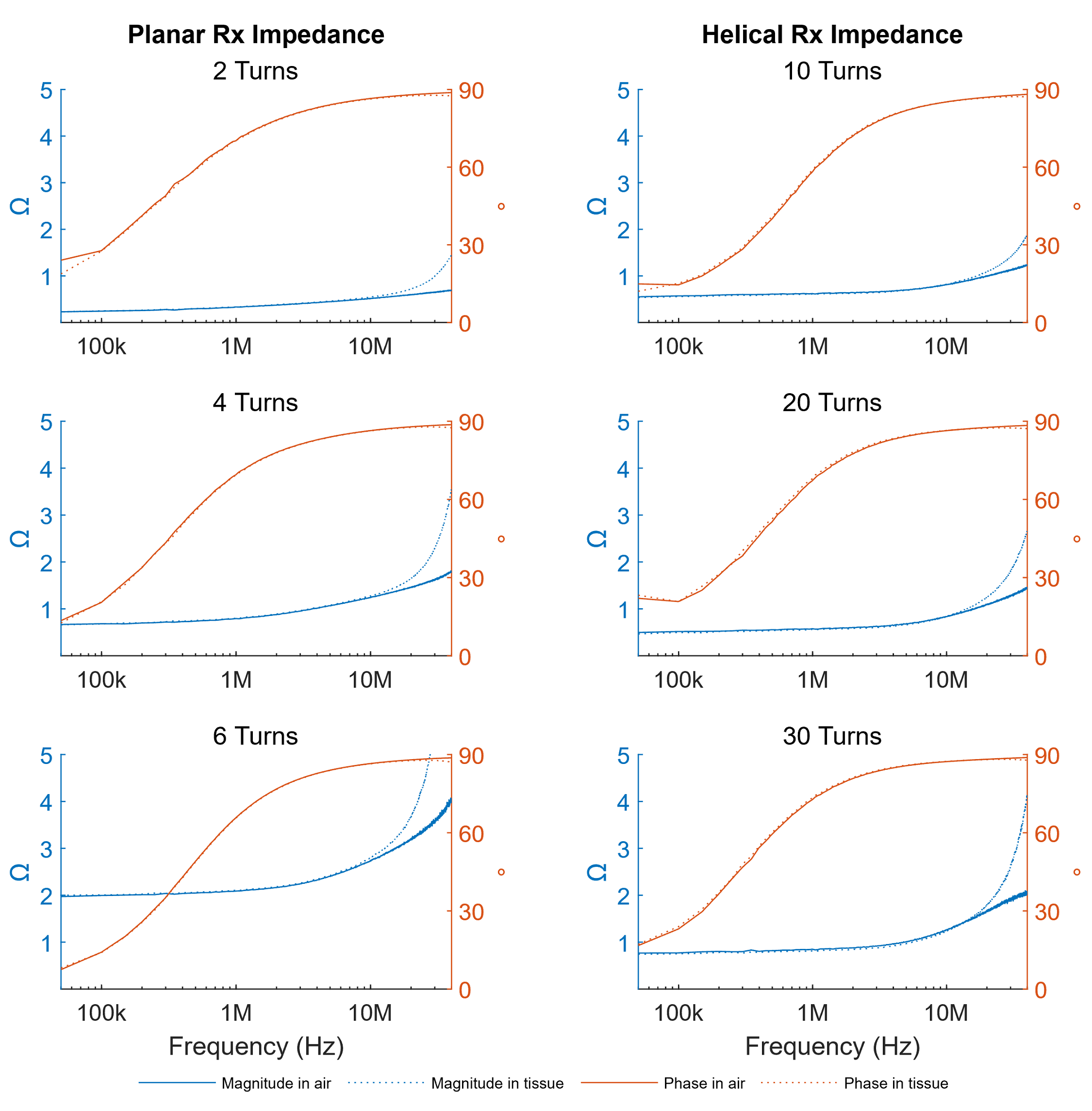

Supplement: S1 Fig — Blue lines show their magnitudes and orange lines show their phases in air (solid lines) or in tissue (dashed lines). Phases are aligned in air and in tissues, but magnitude increases in tissues beyond 10 MHz. (TIF) [file pone.0351138.s001.tif]

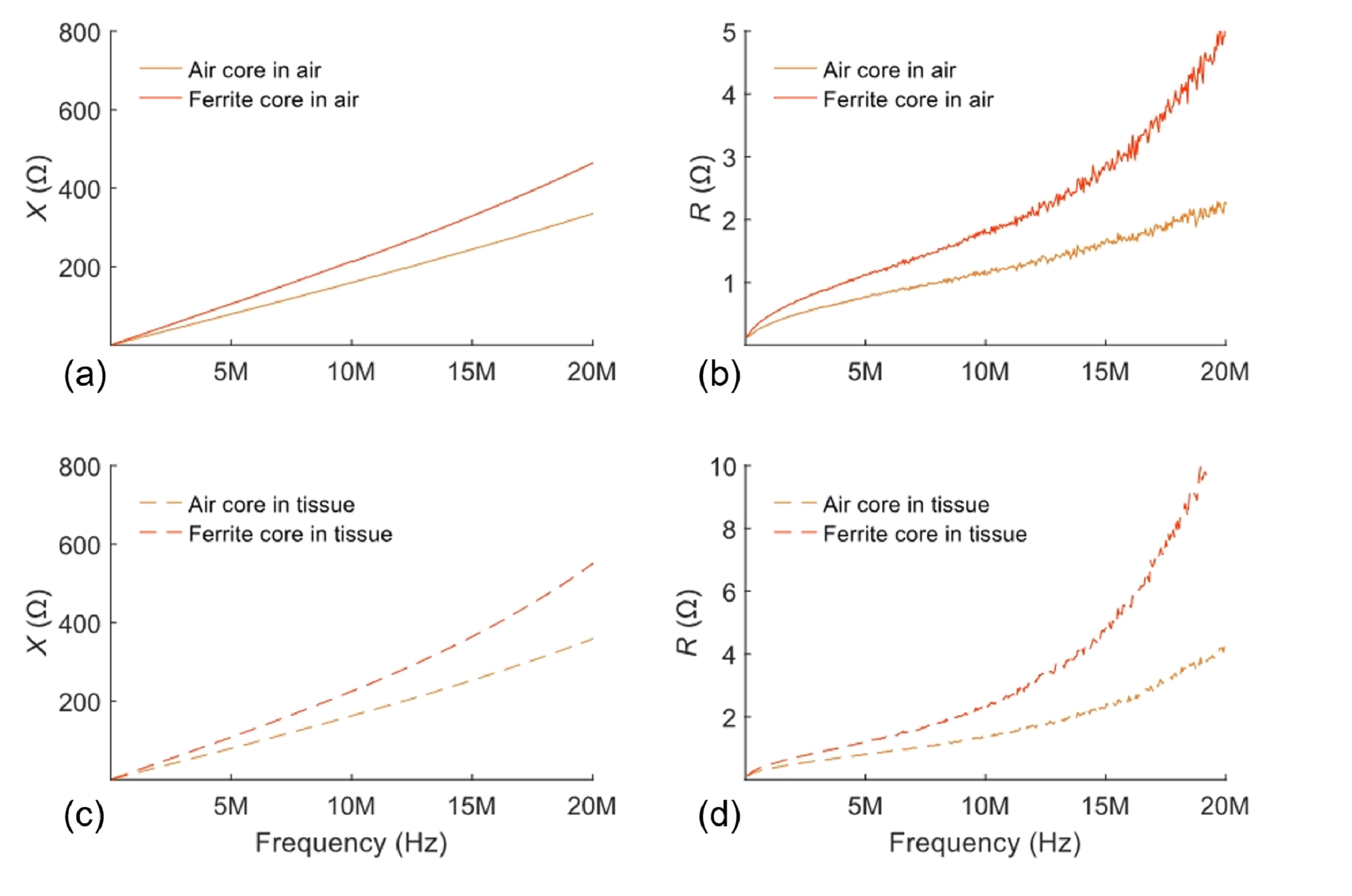

Supplement: S2 Fig — (a) Reactances (X=ω×L) in air. (b) Resistances (R) in air. (c) Reactances in tissue. (d) Resistances in tissue. Ferrite increased X by approximately 30% (a, c), but it introduced a larger increase in R (b, d), particularly in frequency beyond 10 MHz, matching the frequency range where the imaginary permeability starts to increase according to [47]. (TIF) [file pone.0351138.s002.tif]

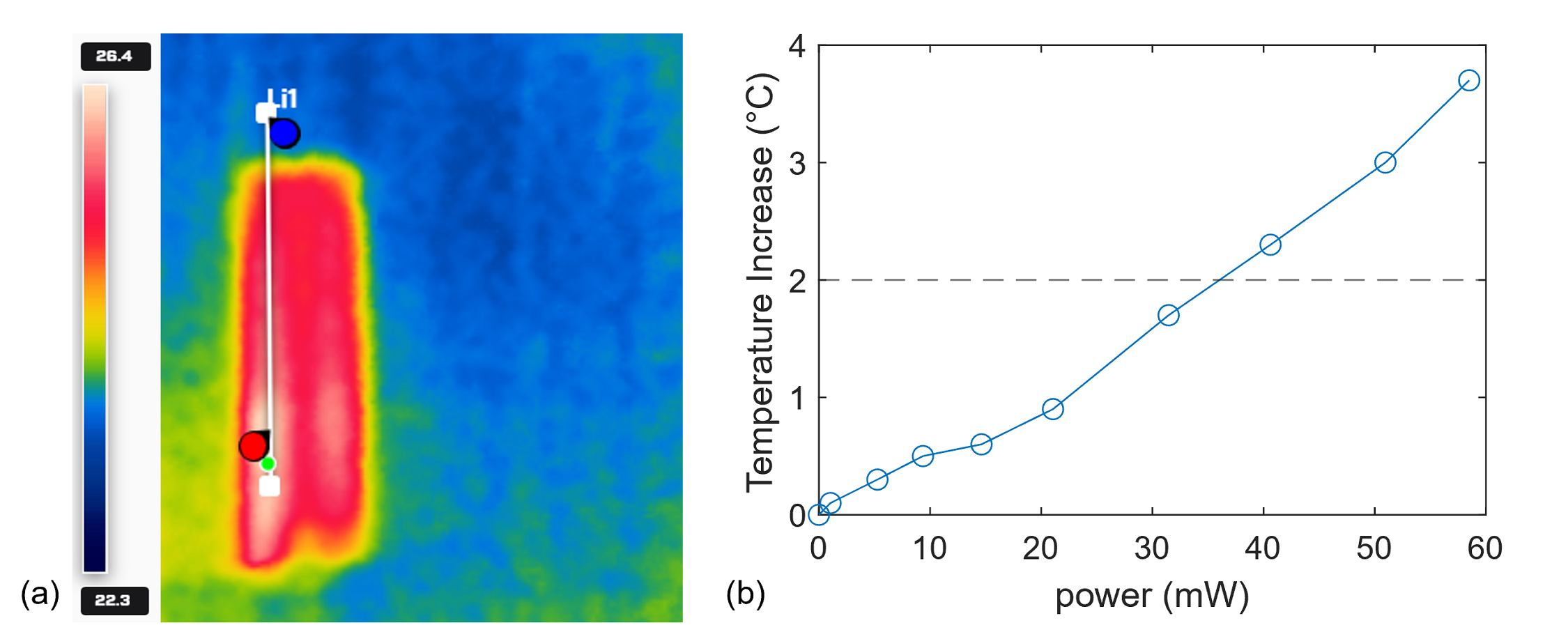

Supplement: S3 Fig — (a) The temperatures were captured by FLIR ETS320 thermal camera. The coil received different amounts of power and its temperature (red mark) was measured at a steady state and compared to surroundings (blue mark). The temperature was recorded from the pixel of the red and blue marks. (b) The coil temperature increase reached 2 °C at 38 mW dissipation, similar to the reported value of 40 mW from [55]. (TIF) [file pone.0351138.s003.tif]
