# Supplementary material for: Toward a fully wireless endovascular neural interface: Evaluating power transfer efficacy
Source: PLoS One. 2026 Jun 15;21(6):e0351138. doi: 10.1371/journal.pone.0351138 (PMC13268161; doi:10.1371/journal.pone.0351138)
Supplement: Attachment — Submitted filename: Responses to Reviewers.docx [file pone.0351138.s007.docx]

**Response to Reviewers**

| **Reviewer 1 comment** | **Response** | **Change made to paper** |
| --- | --- | --- |
| The sharp decrease in PTE from 11% at 15 mm to ~2% at 30 mm *suggests* strong sensitivity to coupling, implantation depth, and anatomical variability, potentially limiting practical ENI deployment. | We agree with the comment and have added brief discussion emphasizing the impact of depth and anatomical limitations. | Lines 359-362. “However, PTE in this study decreased sharply to 2% at the implantation depth of 30 mm, suggesting strong sensitivity of wireless coupling efficiency to implantation depth, anatomical variability, and coil alignment, posing challenges in practical ENI deployment.” |
| Operation at 6.78 and 13.56 MHz relies on high-Q tissue-loaded resonance, which reduces robustness to detuning caused by motion, tissue heterogeneity, or coil deformation. | We agree with the comment and have added discussion about the detuning issue that can occur. | Lines 383-385. “Furthermore, operation within frequency bands requires coil and circuit tuning to the resonance frequency, which could suffer from detuning effects caused by physiological motion, tissues heterogeneity, and coil deformation following implantation.” |
| Although SAR-compliant delivery of 72 mW is reported, SAR constraints fundamentally cap transmitter power, limiting scalability for higher-power or multichannel ENI applications. | We agree with the comment and have added discussion about scalability and multichannel solutions. | Lines 386-389. “This study addressed the upper power limit of 72 mW to wireless endovascular devices based on SAR constraint simulation, serving as a constraint for scalability or high-power applications. Nevertheless, since one channel requires only a few μW for recording and less than 10 mW for stimulation [24], the budget is still sufficient to support multichannel ENI application.” |
| Claims regarding multichannel feasibility are based on single-receiver or equivalent-load models. Mutual coupling and inter-channel interference effects are not evaluated. | We have added additional text discussing expansion to a wireless multichannel device and potential interference effects. | Lines 411-414. “Moreover, a single-receiver, single-load configuration was employed in this scheme. In scenarios involving multiple receivers for simultaneously operating multiple devices or multiple channels, mutual coupling and inter-channel interference should be considered.” |
| The in vivo component of the study is underdeveloped, lacking sufficient methodological detail to assess relevance, safety, and long-term feasibility. Without clearer in vivo validation, the translational implications of the proposed system remain speculative. | The present study focussed on the development of the device and feasibility. However, future studies will focus on efficacy showing safety and long-term feasibility in the future. We have added additional text in the discussion. | Lines 428-433. “This study demonstrated proof of concept for wireless powering of endovascular multichannel ENIs and the outlined quantitative performance metrics for power efficiency and power budgets. Future work will focus on the development of a fully implantable system incorporating integrated circuitry and electrodes, as well as the evaluation of long-term device stability and biological tissue safety, with the ultimate goal of translation to clinical applications.” |
| Despite improvements with the flux-pipe coil, performance remains sensitive to angular and lateral misalignment, which may be problematic in dynamic in vivo environments. | We have added further text in the discussion to discuss the results emphasising the impact of misalignment. | Lines 409-410. “The misalignments could reduce the PTE from its initial estimation in dynamic in vivo environments.” |
| SAR and power delivery estimates are largely computational and may not fully capture complex in vivo thermal and electromagnetic effects. | We agree with the limitations and have added further text to discuss this. | Lines 395-397. “Since the computational model may not fully capture the complex thermal and electromagnetic interaction in vivo, future studies should attempt to measure actual tissue heating using in-vivo studies.” |

| **Reviewer 2 comment** | **Response** | **Change made to paper** |
| --- | --- | --- |
| Numerous studies have reported WPT coil designs for implants. The specific advantages of the proposed planar, flux-pipe and figure-8 transmitters over existing literature (e.g., works cited in Table 1) are not sufficiently quantified. Furthermore, the rationale for designing the planar Rx coil as a rectangle lacks a deep theoretical or experimental basis.  The authors are encouraged to add parametric comparisons (e.g., radar plot) showing the advantages of the three design. | We have added text to justify the use of rectangular instead of planar coil as Rx and highlight the fact that work in Table 1 lacks Tx coil design. Since these coils operate in inductive near-fields, it may not be suitable to use radar plots. | Lines 88-90. “These devices were primarily designed for temporary hemodynamic monitoring or hyperthermia pacing applications, rather than long-term neural interfaces, so they lack a wearable and compact transmitter (Tx) coil design.”  Lines 179-184. “In the context of ENIs, the stent itself is not required to maintain patency of the vessel or mechanical support. Theoretically, a rectangular coil, of the same size of a stent, could be used as the primary structure, thereby increasing the effective coil size to capture magnetic flux and improve the receiver efficiency.” |
| It seems weird as the ferrite core reduces Qtx in the planar coil. Please explain possible reasons. | Further text has been added to elaborate the possible reasons. | Lines 235-239. “The observed reduction of $Q$ with the inclusion of ferrite can be attributed to a disproportionate increase in resistance relative to inductance. Although the ferrite increased the coil inductance by approximately 30%, it introduced a larger increase in total resistance. This additional resistance is likely due to the eddy current or hysteresis loss present in the ferrites at high frequencies [46].” |
| Please also provide internal properties of Rx coils, like impedance and phase versus frequency, for further verification. | We have provided more coil properties in Supplementary Figure 1 | Lines 644-648. “Fig S1. Impedance of all Rx coils (planar and helical with different turns). Blue lines show their magnitudes and orange lines show their phases in air (solid lines) or in tissue (dashed lines). Phases are aligned in air and in tissue, but magnitude increases in tissue beyond 10 MHz.” |
| As frequency is critical in wireless power transfer, please clarify the conditions in all measurements. | We have clarified in each figure. | Line 214. “Wireless link simulation and measurement at 10 MHz.”  Line 277. Updated figure.  Line 296. Updated figure.  Line 297. “SAR simulations for the three coil pairs at each’s operational frequency.”  Line 323. Updated figure. Line 324. “Misalignment investigations for the three coil pairs at each’s operational frequency.”  Line 343. Updated figure.  Lines 344-348. “Load matching simulation with different coil turns. (a) Rx coil prototypes with 2, 4, and 6 turns for planar Rx and 10, 20, 30 turns for helical Rx. Load resistance versus PDL at 13.56 MHz (b) for different turns of planar Rx with planar Tx, (c) for different turns of helical Rx with flux-pipe Tx at 6.78 MHz, and (d) for different turns of helical Rx with figure-8 Tx at 13.56 MHz. The power source was set to 500 mW and the inter-coil distance was fixed to 20 mm.” |
| The Rx coils presented have a diameter of 3 mm, which may be prohibitive for many intracranial vessels. How does the PTE decay if the coil is miniaturized to fit smaller vascular? Please provide theoretical or simulation-based evidence (e.g., a systematic analysis of scaling laws) to support the usability in real vascular dimension. | We agree the usability in different vascular dimension is important and have added quantitative scaling laws limitations and discussion of the solutions. | Lines 415-427. “Recent work has demonstrated the use of an endovascular neural interface in submillimeter-scale vasculature to achieve single-cell resolutions [57]. However, miniaturizing Rx coils to accommodate smaller vessels leads to substantial efficiency reductions. In previous investigation, a reduction of Rx coil by 4 times in length decreased PTE more than tenfold at 10 mm implant depth, and more than 60 times at 30 mm implant depth [58]. This depth-dependent degradation indicates that efficiency loss becomes increasingly severe at deeper implantation sites, where smaller vessels are usually located. Furthermore, since these vessels are typically oriented perpendicular, not parallel, to the scalp surface, their endovascular coils require different coil geometric design. Nevertheless, the cerebral vascular networks are interconnected. Therefore, we propose that the coils designed in this study can still be implanted in SSS and wire-connected to smaller electrode arrays in smaller or deeper vessels. This hybrid approach may help preserve wireless link strength while requiring minimal lead length.” |
| The endovascular environment involves blood flow and potential biological deposition, which may alter coil impedance and quality factors.  The authors should include testing in saline or blood-mimicking environments to characterize the impact of biological loading on resonance frequency and efficiency. Additional test on biocompatibility of the implants is also recommended. | We have filled the tubes containing the coil with saline during tissue measurement. We have clarified this in the Method section. | Line 164-165. “The Rx coils were inserted into plastic tubes of 3-mm diameter filled with saline.” |
| Please also measure temperature change to further justify the safety of proposed systems. | Previous work has verified that power consumption up to tens of milliwatts can be safely operated in brain implantable devices. We have added additional text discussing these points. | Lines 392-397. “Previous studies have shown that a power dissipation of 40 mW from brain implants can cause a local temperature elevation of 2 °C [56]. For endovascular devices, heating issues are expected to be mitigated by the cooling effect of blood flow. Since the computational model may not fully capture the complex thermal and electromagnetic interaction in vivo, future studies should attempt to measure actual tissue heating using in-vivo studies.” |
| The characterization of rotational and tilting misalignments in Figure 6 appears oversimplified. It is recommended to present more results reflecting the combined effects of lateral displacement and angular tilt. Stability should also be discussed in the context of realistic anatomical vessel curvatures (e.g., S-shaped segments). | The external Tx coil will be placed at a location of maximum wireless link strength, so lateral displacement misalignments will be eliminated in this application and thus were not measured. We have added text in the discussion on this point. | Lines 403-405. “Lateral misalignments can be mitigated by repositioning the external Tx coil to align with the implanted Rx coil and achieve highest link strength, so they were excluded from this study.” |
| The abstract mentions electrodes and in vivo results, yet no such data appear in the manuscript. For a device presented as a neural interface, the manuscript lacks essential electrochemical characterizations of the electrodes and fails to show any actual neural recording or stimulation waveform. Moreover, the data in Figures 3 through 7 appear to rely solely on FEA simulations or bench-top measurements. No evidence from the living biological environment is provided. The authors should either remove these claims from the abstract or provide the corresponding experimental results. | We have updated figures to correctly label in vivo data. We did not mention or claim electrode use in this work. While a neural interface is the intended application, we focus on investigating the underlying wireless power transfer. We have added additional text discussing these points. | Line 214. Updated figure.  Line 277. Updated figure.  Line 323. Updated figure.  Lines 428-433. “This study demonstrated proof of concept for wireless powering of endovascular multichannel ENIs and the outlined quantitative performance metrics for power efficiency and power budgets. Future work will focus on the development of a fully implantable system incorporating integrated circuitry and electrodes, as well as the evaluation of long-term device stability and biological tissue safety, with the ultimate goal of translation to clinical applications.” |
| In figure3 g,h,i, please also label the figure legends properly. | The figure legend has now been labelled | Line 250. Updated figure. |
| In figure4, it is more fair to measure PTE under the same conditions. | We have added text to highlight this. | Lines 299-300. “All conditions were the same except for the operating frequencies were chosen for the highest efficiency for each coil pair.” |
| In figure5 b,c, please label the figure legends. | The figure legend has now been labelled | Line 296. Updated figure. |
| In Figure 6b, please also provide labels. | We have added the labels to figure 6b | Line 323. Updated figure. |
| In Figure 7, please clarify the measurement condition, especially the working frequency. | We have added information in the caption and the associated text. | Line 343. Updated figure.  Lines 344-348 “Load matching simulation with different coil turns. (a) Rx coil prototypes with 2, 4, and 6 turns for planar Rx and 10, 20, 30 turns for helical Rx. Load resistance versus PDL at 13.56 MHz (b) for different turns of planar Rx with planar Tx, (c) for different turns of helical Rx with flux-pipe Tx at 6.78 MHz, and (d) for different turns of helical Rx with figure-8 Tx at 13.56 MHz. The power source was set to 500 mW and the inter-coil distance was fixed to 20 mm.” |
