# Supplementary material for: Toward a fully wireless endovascular neural interface: Evaluating power transfer efficacy
Source: PLoS One. 2026 Jun 15;21(6):e0351138. doi: 10.1371/journal.pone.0351138 (PMC13268161; doi:10.1371/journal.pone.0351138)
Supplement: Attachment — Submitted filename: Responses to Reviewers (2).docx [file pone.0351138.s008.docx]

**Response to Reviewers**

| **Reviewer 1 comment** | **Response** | **Change made to paper** |
| --- | --- | --- |
| The authors have carefully addressed all previously raised concerns. The revised version is clearer and demonstrates a notable improvement in overall quality. | We appreciate the reviewer’s comment that help us improve the manuscript. | N/A |

| **Reviewer 2 comment** | **Response** | **Change made to paper** |
| --- | --- | --- |
| Regarding my previous comment 1.1, radar plot means a graph to compare different metrics of the authors’ coil and other coils reported in the literature. It has nothing to do with near-field or far-field. | We misunderstand the comment in the previous round, and have now added a radar plot. | Lines 104-105. “Fig 2 compares the design requirements for the aforementioned endovascular devices.”  Lines 106 “Fig 2. The design requirements for different types of endovascular devices.” |
| Regarding my previous comment 1.2, it’s still not clear why the ferrite core reduces Qtx. The authors added some hypothesis but did not provide evidence, such as experimental test, to prove it. | We included the experimental results in Fig S2 and added evidence from the datasheet of the ferrite material. | Lines 236-240. “Although the ferrite increased the coil inductance by approximately 30% (Figs S2a, c), it introduced a larger increase in total resistance (Fig S2b, d), and this increase dominates over the inductance enhancement, resulting in a net reduction in $Q_{\mathrm{Tx}}$. This additional resistance is likely due to the eddy current or hysteresis loss present in the ferrites at high frequencies [45], as well as the imaginary permeability of the ferrite, will results in internal magnetic loss, according to the ferrite material datasheet [47].”  Lines 666-669. “Fig S2. Impedance of the planar Tx coil. (a) Reactances (X=ω×L) in air. (b) Resistances (R) in air. (c) Reactances in tissue. (d) Resistances in tissue. Ferrite increased X by approximately 30% (a, c), but it introduced a larger increase in R (b, d), particularly in frequency beyond 10 MHz, matching the frequency range where the imaginary permeability starts to increase according to [47].” |
| Regarding my previous comment 3.2, the authors responded by citing a literature and mentioning the inaccuracy in modeling. However, results from other literatures cannot reflect the temperature change of the authors’ coil. The authors can directly measure the temperature change experimentally without computational model | We conducted the measurement and added the results in Fig S3. | Line 389. “Our measurement of coil heating yielded similar results (Fig S3).”  Lines 670-674. “Fig S3. Measurement of temperature increase of the Rx coil vs. its power dissipation. (a) The temperatures were captured by FLIR ETS320 thermal camera. The coil received different amounts of power and its temperature (red mark) was measured at a steady state and compared to surroundings (blue mark). The temperature was recorded from the pixel of the red and blue marks. (b) The coil temperature increase reached 2 °C at 38 mW dissipation, similar to the reported value of 40 mW from [55].” |
