# Supplementary material for: Toward a fully wireless endovascular neural interface: Evaluating power transfer efficacy
Source: PLoS One. 2026 Jun 15;21(6):e0351138. doi: 10.1371/journal.pone.0351138 (PMC13268161; doi:10.1371/journal.pone.0351138)
Supplement: Peer review history

# Peer review: Decision Letter 0

**DOI:** 10.1371/journal.pone.0351138.r001

**Authors:** Baeckkyoung Sung

16 Jan 2026

PONE-D-25-58465

Toward a Fully Wireless Endovascular Neural Interface: Evaluating Power Transfer Efficacy

PLOS One

Dear Dr. Tai,

Thank you for submitting your manuscript to PLOS ONE. After careful consideration, we feel that it has merit but does not fully meet PLOS ONE’s publication criteria as it currently stands. Therefore, we invite you to submit a revised version of the manuscript that addresses the points raised during the review process.

Please submit your revised manuscript by Mar 02 2026 11:59PM. If you will need more time than this to complete your revisions, please reply to this message or contact the journal office at plosone@plos.org. When you're ready to submit your revision, log on to https://www.editorialmanager.com/pone/ and select the 'Submissions Needing Revision' folder to locate your manuscript file.

Please include the following items when submitting your revised manuscript:

- A letter that responds to each point raised by the academic editor and reviewer(s). You should upload this letter as a separate file labeled 'Response to Reviewers'.
- A marked-up copy of your manuscript that highlights changes made to the original version. You should upload this as a separate file labeled 'Revised Manuscript with Track Changes'.
- An unmarked version of your revised paper without tracked changes. You should upload this as a separate file labeled 'Manuscript'.

If you would like to make changes to your financial disclosure, please include your updated statement in your cover letter. Guidelines for resubmitting your figure files are available below the reviewer comments at the end of this letter.

If applicable, we recommend that you deposit your laboratory protocols in protocols.io to enhance the reproducibility of your results. Protocols.io assigns your protocol its own identifier (DOI) so that it can be cited independently in the future. For instructions see: https://journals.plos.org/plosone/s/submission-guidelines#loc-laboratory-protocols. Additionally, PLOS ONE offers an option for publishing peer-reviewed Lab Protocol articles, which describe protocols hosted on protocols.io. Read more information on sharing protocols at https://plos.org/protocols?utm_medium=editorial-email&utm_source=authorletters&utm_campaign=protocols.

We look forward to receiving your revised manuscript.

Kind regards,

Baeckkyoung Sung, Ph.D.

Academic Editor

PLOS One

Journal Requirements:

When submitting your revision, we need you to address these additional requirements.

1. Please ensure that your manuscript meets PLOS ONE's style requirements, including those for file naming. The PLOS ONE style templates can be found at

https://journals.plos.org/plosone/s/file?id=wjVg/PLOSOne_formatting_sample_main_body.pdf and

https://journals.plos.org/plosone/s/file?id=ba62/PLOSOne_formatting_sample_title_authors_affiliations.pdf

2. In your Methods section, please report the source of the animals (sheep) and include a comment about the state of the animals following this research. Were they euthanized or housed for use in further research? If any animals were sacrificed by the authors, please include the method of euthanasia and describe any efforts that were undertaken to reduce animal suffering."

3. Please note that PLOS One has specific guidelines on code sharing for submissions in which author-generated code underpins the findings in the manuscript. In these cases, we expect all author-generated code to be made available without restrictions upon publication of the work. Please review our guidelines at https://journals.plos.org/plosone/s/materials-and-software-sharing#loc-sharing-code and ensure that your code is shared in a way that follows best practice and facilitates reproducibility and reuse.

4. Thank you for stating the following financial disclosure:

“This research is supported by National Health and Medical Research Council, Australia (1158912 & 2000153) and U.S. Department of Defense, Epilepsy Research Program CDMRP (EP170058 & EP210052).”

Please state what role the funders took in the study.  If the funders had no role, please state: "The funders had no role in study design, data collection and analysis, decision to publish, or preparation of the manuscript."

If this statement is not correct you must amend it as needed.

Please include this amended Role of Funder statement in your cover letter; we will change the online submission form on your behalf.

5. Thank you for stating the following in the Competing Interests section:

“DBG and SEJ have patents related to the Stentrode™ technology similar to the device used in this study. SEJ was previously employed at Synchron Australia (previously SmartStent Pty Ltd) and DBG was previously a member of the Scientific Advisory Committee of Synchron Inc. DBG is the Director of and SEJ is an investigator in the ARC Training Centre in Cognitive Computing for Medical Technologies for research in brain-computer interface technologies (ARC CCMT); Synchron Inc. is a partner providing co-funding and in-kind support to the ARC CCMT. The work described in this manuscript was not funded or supported by Synchron Inc.”

Please confirm that this does not alter your adherence to all PLOS ONE policies on sharing data and materials, by including the following statement: "This does not alter our adherence to  PLOS ONE policies on sharing data and materials.” (as detailed online in our guide for authors http://journals.plos.org/plosone/s/competing-interests).  If there are restrictions on sharing of data and/or materials, please state these. Please note that we cannot proceed with consideration of your article until this information has been declared.

Please include your updated Competing Interests statement in your cover letter; we will change the online submission form on your behalf.

6. When completing the data availability statement of the submission form, you indicated that you will make your data available on acceptance. We strongly recommend all authors decide on a data sharing plan before acceptance, as the process can be lengthy and hold up publication timelines. Please note that, though access restrictions are acceptable now, your entire data will need to be made freely accessible if your manuscript is accepted for publication. This policy applies to all data except where public deposition would breach compliance with the protocol approved by your research ethics board. If you are unable to adhere to our open data policy, please kindly revise your statement to explain your reasoning and we will seek the editor's input on an exemption. Please be assured that, once you have provided your new statement, the assessment of your exemption will not hold up the peer review process

7. If the reviewer comments include a recommendation to cite specific previously published works, please review and evaluate these publications to determine whether they are relevant and should be cited. There is no requirement to cite these works unless the editor has indicated otherwise.

Additional Editor Comments:

The manuscript needs to be revised in accordance to the reviewers' comments.

[Note: HTML markup is below. Please do not edit.]

Reviewers' comments:

Reviewer's Responses to Questions

**Comments to the Author**

1. Is the manuscript technically sound, and do the data support the conclusions?

The manuscript must describe a technically sound piece of scientific research with data that supports the conclusions. Experiments must have been conducted rigorously, with appropriate controls, replication, and sample sizes. The conclusions must be drawn appropriately based on the data presented.

Reviewer #1: Yes

Reviewer #2: Yes

**********

2. Has the statistical analysis been performed appropriately and rigorously?

Reviewer #1: Yes

Reviewer #2: Yes

**********

3. Have the authors made all data underlying the findings in their manuscript fully available?

The PLOS Data policy requires authors to make all data underlying the findings described in their manuscript fully available without restriction, with rare exception (please refer to the Data Availability Statement in the manuscript PDF file). The data should be provided as part of the manuscript or its supporting information, or deposited to a public repository. For example, in addition to summary statistics, the data points behind means, medians and variance measures should be available. If there are restrictions on publicly sharing data—e.g. participant privacy or use of data from a third party—those must be specified.

Reviewer #1: Yes

Reviewer #2: Yes

**********

4. Is the manuscript presented in an intelligible fashion and written in standard English?

PLOS ONE does not copyedit accepted manuscripts, so the language in submitted articles must be clear, correct, and unambiguous. Any typographical or grammatical errors should be corrected at revision, so please note any specific errors here.

Reviewer #1: Yes

Reviewer #2: Yes

**********

5. Review Comments to the Author

Please use the space provided to explain your answers to the questions above. You may also include additional comments for the author, including concerns about dual publication, research ethics, or publication ethics. (Please upload your review as an attachment if it exceeds 20,000 characters)

Reviewer #1: The manuscript “Toward a Fully Wireless Endovascular Neural Interface: Evaluating Power Transfer Efficacy” investigates the performance of three distinct wireless transmitter (Tx) coil designs coupled to receivers for endovascular neural interface (ENI) applications. The Tx configurations include a rectangular planar coil adapted from conventional wearable square or circular coils, a flux-pipe coil inspired by electric vehicle charging systems, and a figure-8 coil derived from transcranial magnetic stimulation designs. The authors systematically evaluate optimal operating frequency, transmitter–receiver coupling, power transfer efficiency, specific absorption rate (SAR), misalignment tolerance, and load mismatch in both air and tissue models. The study further examines system performance with and without ferrite materials.

Overall, the device characterization is thorough and well executed, combining theoretical analysis, computational modeling, and experimental validation. The results indicate improved operational efficiency for the rectangular planar coil under SAR safety constraints. The work represents a valuable contribution to the development of wireless power transfer strategies for ENIs. Below are specific comments and concerns.

Major Concerns

The sharp decrease in PTE from 11% at 15 mm to ~2% at 30 mm suggests strong sensitivity to coupling, implantation depth, and anatomical variability, potentially limiting practical ENI deployment.

Operation at 6.78 and 13.56 MHz relies on high-Q tissue-loaded resonance, which reduces robustness to detuning caused by motion, tissue heterogeneity, or coil deformation.

Although SAR-compliant delivery of 72 mW is reported, SAR constraints fundamentally cap transmitter power, limiting scalability for higher-power or multichannel ENI applications.

Claims regarding multichannel feasibility are based on single-receiver or equivalent-load models. Mutual coupling and inter-channel interference effects are not evaluated.

The in vivo component of the study is underdeveloped, lacking sufficient methodological detail to assess relevance, safety, and long-term feasibility. Without clearer in vivo validation, the translational implications of the proposed system remain speculative.

Minor Concerns

Despite improvements with the flux-pipe coil, performance remains sensitive to angular and lateral misalignment, which may be problematic in dynamic in vivo environments.

SAR and power delivery estimates are largely computational and may not fully capture complex in vivo thermal and electromagnetic effects.

Reviewer #2: The manuscript presents the significance of eliminating wires for the endovascular interface and performs characterization of wireless power transfer schemes using three different designs, though the working principle is not new. The authors conduct finite element simulation and bench-top measurements to characterize coupling coefficients, power transfer efficiency, misalignment tolerance, and SAR limits. However, the following major comments must be addressed before reconsideration for publication in PLOS ONE.

1. Numerous studies have reported WPT coil designs for implants. The specific advantages of the proposed planar, flux-pipe and figure-8 transmitters over existing literature (e.g., works cited in Table 1) are not sufficiently quantified. Furthermore, the rationale for designing the planar Rx coil as a rectangle lacks a deep theoretical or experimental basis.

(1) The authors are encouraged to add parametric comparisons (e.g., radar plot) showing the advantages of the three design.

(2) It seems weird as the ferrite core reduces Qtx in the planar coil. Please explain possible reasons.

(3) Please also provide internal properties of Rx coils, like impedance and phase versus frequency, for further verification.

(4) As frequency is critical in wireless power transfer, please clarify the conditions in all measurements.

2. The Rx coils presented have a diameter of 3 mm, which may be prohibitive for many intracranial vessels. How does the PTE decay if the coil is miniaturized to fit smaller vascular? Please provide theoretical or simulation-based evidence (e.g., a systematic analysis of scaling laws) to support the usability in real vascular dimension.

3. The endovascular environment involves blood flow and potential biological deposition, which may alter coil impedance and quality factors.

(1) The authors should include testing in saline or blood-mimicking environments to characterize the impact of biological loading on resonance frequency and efficiency. Additional test on biocompatibility of the implants is also recommended.

(2) Please also measure temperature change to further justify the safety of proposed systems.

4. The characterization of rotational and tilting misalignments in Figure 6 appears oversimplified. It is recommended to present more results reflecting the combined effects of lateral displacement and angular tilt. Stability should also be discussed in the context of realistic anatomical vessel curvatures (e.g., S-shaped segments).

5. The abstract mentions electrodes and in vivo results, yet no such data appear in the manuscript. For a device presented as a neural interface, the manuscript lacks essential electrochemical characterizations of the electrodes and fails to show any actual neural recording or stimulation waveform. Moreover, the data in Figures 3 through 7 appear to rely solely on FEA simulations or bench-top measurements. No evidence from the living biological environment is provided. The authors should either remove these claims from the abstract or provide the corresponding experimental results.

6. Some details in the figures that are not clearly explained also require further revision

(1) In figure3 g,h,i, please also label the figure legends properly.

(2) In figure4, it is more fair to measure PTE under the same conditions.

(3) In figure5 b,c, please label the figure legends.

(4) In Figure 6b, please also provide labels.

(5) In Figure 7, please clarify the measurement condition, especially the working frequency.

**********

6. PLOS authors have the option to publish the peer review history of their article (what does this mean?). If published, this will include your full peer review and any attached files.

If you choose “no”, your identity will remain anonymous but your review may still be made public.

**Do you want your identity to be public for this peer review?**  For information about this choice, including consent withdrawal, please see our Privacy Policy.

Reviewer #1: No

Reviewer #2: No

**********

[NOTE: If reviewer comments were submitted as an attachment file, they will be attached to this email and accessible via the submission site. Please log into your account, locate the manuscript record, and check for the action link "View Attachments". If this link does not appear, there are no attachment files.]

To ensure your figures meet our technical requirements, please review our figure guidelines: https://journals.plos.org/plosone/s/figures

You may also use PLOS’s free figure tool, NAAS, to help you prepare publication quality figures: https://journals.plos.org/plosone/s/figures#loc-tools-for-figure-preparation.

NAAS will assess whether your figures meet our technical requirements by comparing each figure against our figure specifications.

# Author comment: Author response to Decision Letter 1

**DOI:** 10.1371/journal.pone.0351138.r002

24 Feb 2026

Thank you for the reviews and comments. We have addressed your comments in the "Responses to Reviewers" document.

Attachment Submitted filename: Responses to Reviewers.docx

# Peer review: Decision Letter 1

**DOI:** 10.1371/journal.pone.0351138.r003

**Authors:** Baeckkyoung Sung

18 Mar 2026

PONE-D-25-58465R1

Toward a Fully Wireless Endovascular Neural Interface: Evaluating Power Transfer Efficacy

PLOS One

Dear Dr. Tai,

Thank you for submitting your manuscript to PLOS ONE. After careful consideration, we feel that it has merit but does not fully meet PLOS ONE’s publication criteria as it currently stands. Therefore, we invite you to submit a revised version of the manuscript that addresses the points raised during the review process.

Please submit your revised manuscript by May 02 2026 11:59PM. If you will need more time than this to complete your revisions, please reply to this message or contact the journal office at plosone@plos.org. When you're ready to submit your revision, log on to https://www.editorialmanager.com/pone/ and select the 'Submissions Needing Revision' folder to locate your manuscript file.

Please include the following items when submitting your revised manuscript:

- A letter that responds to each point raised by the academic editor and reviewer(s). You should upload this letter as a separate file labeled 'Response to Reviewers'.
- A marked-up copy of your manuscript that highlights changes made to the original version. You should upload this as a separate file labeled 'Revised Manuscript with Track Changes'.
- An unmarked version of your revised paper without tracked changes. You should upload this as a separate file labeled 'Manuscript'.

-->If you would like to make changes to your financial disclosure, please include your updated statement in your cover letter. Guidelines for resubmitting your figure files are available below the reviewer comments at the end of this letter.

If applicable, we recommend that you deposit your laboratory protocols in protocols.io to enhance the reproducibility of your results. Protocols.io assigns your protocol its own identifier (DOI) so that it can be cited independently in the future. For instructions see: https://journals.plos.org/plosone/s/submission-guidelines#loc-laboratory-protocols. Additionally, PLOS ONE offers an option for publishing peer-reviewed Lab Protocol articles, which describe protocols hosted on protocols.io. Read more information on sharing protocols at https://plos.org/protocols?utm_medium=editorial-email&utm_source=authorletters&utm_campaign=protocols.

We look forward to receiving your revised manuscript.

Kind regards,

Baeckkyoung Sung, Ph.D.

Academic Editor

PLOS One

Journal Requirements:

If the reviewer comments include a recommendation to cite specific previously published works, please review and evaluate these publications to determine whether they are relevant and should be cited. There is no requirement to cite these works unless the editor has indicated otherwise.

Please review your reference list to ensure that it is complete and correct. If you have cited papers that have been retracted, please include the rationale for doing so in the manuscript text, or remove these references and replace them with relevant current references. Any changes to the reference list should be mentioned in the rebuttal letter that accompanies your revised manuscript. If you need to cite a retracted article, indicate the article’s retracted status in the References list and also include a citation and full reference for the retraction notice.

Additional Editor Comments (if provided):

The authors are recommended to revise the manuscript based on the feedback by Reviewer #2.

[Note: HTML markup is below. Please do not edit.]

Reviewers' comments:

Reviewer's Responses to Questions

**Comments to the Author**

1. If the authors have adequately addressed your comments raised in a previous round of review and you feel that this manuscript is now acceptable for publication, you may indicate that here to bypass the “Comments to the Author” section, enter your conflict of interest statement in the “Confidential to Editor” section, and submit your "Accept" recommendation.

Reviewer #1: All comments have been addressed

Reviewer #2: (No Response)

**********

2. Is the manuscript technically sound, and do the data support the conclusions?

The manuscript must describe a technically sound piece of scientific research with data that supports the conclusions. Experiments must have been conducted rigorously, with appropriate controls, replication, and sample sizes. The conclusions must be drawn appropriately based on the data presented.

Reviewer #1: Yes

Reviewer #2: (No Response)

**********

3. Has the statistical analysis been performed appropriately and rigorously?

Reviewer #1: N/A

Reviewer #2: (No Response)

**********

4. Have the authors made all data underlying the findings in their manuscript fully available?

The PLOS Data policy requires authors to make all data underlying the findings described in their manuscript fully available without restriction, with rare exception (please refer to the Data Availability Statement in the manuscript PDF file). The data should be provided as part of the manuscript or its supporting information, or deposited to a public repository. For example, in addition to summary statistics, the data points behind means, medians and variance measures should be available. If there are restrictions on publicly sharing data—e.g. participant privacy or use of data from a third party—those must be specified.

Reviewer #1: Yes

Reviewer #2: (No Response)

**********

5. Is the manuscript presented in an intelligible fashion and written in standard English?

PLOS ONE does not copyedit accepted manuscripts, so the language in submitted articles must be clear, correct, and unambiguous. Any typographical or grammatical errors should be corrected at revision, so please note any specific errors here.

Reviewer #1: Yes

Reviewer #2: (No Response)

**********

6. Review Comments to the Author

Please use the space provided to explain your answers to the questions above. You may also include additional comments for the author, including concerns about dual publication, research ethics, or publication ethics. (Please upload your review as an attachment if it exceeds 20,000 characters)

Reviewer #1: The authors have carefully addressed all previously raised concerns. The revised version is clearer and demonstrates a notable improvement in overall quality.

Reviewer #2: Regarding my previous comment 1.1, radar plot means a graph to compare different metrics of the authors’ coil and other coils reported in the literature. It has nothing to do with near-field or far-field.

Regarding my previous comment 1.2, it’s still not clear why the ferrite core reduces Qtx. The authors added some hypothesis but did not provide evidence, such as experimental test, to prove it.

Regarding my previous comment 3.2, the authors responded by citing a literature and mentioning the inaccuracy in modeling. However, results from other literatures cannot reflect the temperature change of the authors’ coil. The authors can directly measure the temperature change experimentally without computational model.

**********

7. PLOS authors have the option to publish the peer review history of their article (what does this mean?). If published, this will include your full peer review and any attached files.

If you choose “no”, your identity will remain anonymous but your review may still be made public.

**Do you want your identity to be public for this peer review?**  For information about this choice, including consent withdrawal, please see our Privacy Policy.

Reviewer #1: No

Reviewer #2: No

**********

[NOTE: If reviewer comments were submitted as an attachment file, they will be attached to this email and accessible via the submission site. Please log into your account, locate the manuscript record, and check for the action link "View Attachments". If this link does not appear, there are no attachment files.]

To ensure your figures meet our technical requirements, please review our figure guidelines: https://journals.plos.org/plosone/s/figures

You may also use PLOS’s free figure tool, NAAS, to help you prepare publication quality figures: https://journals.plos.org/plosone/s/figures#loc-tools-for-figure-preparation.

NAAS will assess whether your figures meet our technical requirements by comparing each figure against our figure specifications.

# Author comment: Author response to Decision Letter 2

**DOI:** 10.1371/journal.pone.0351138.r004

28 Apr 2026

please see the Responses to Reviewers File

Attachment Submitted filename: Responses to Reviewers (2).docx

# Peer review: Decision Letter 2

**DOI:** 10.1371/journal.pone.0351138.r005

**Authors:** Baeckkyoung Sung

24 May 2026

Toward a Fully Wireless Endovascular Neural Interface: Evaluating Power Transfer Efficacy

PONE-D-25-58465R2

Dear Dr. Tai,

We’re pleased to inform you that your manuscript has been judged scientifically suitable for publication and will be formally accepted for publication once it meets all outstanding technical requirements.

Within one week, you’ll receive an e-mail detailing the required amendments. When these have been addressed, you’ll receive a formal acceptance letter and your manuscript will be scheduled for publication.

An invoice will be generated when your article is formally accepted. Please note, if your institution has a publishing partnership with PLOS and your article meets the relevant criteria, all or part of your publication costs will be covered. Please make sure your user information is up-to-date by logging into Editorial Manager at Editorial Manager® and clicking the ‘Update My Information' link at the top of the page. For questions related to billing, please contact billing support.

If your institution or institutions have a press office, please notify them about your upcoming paper to help maximize its impact. If they’ll be preparing press materials, please inform our press team as soon as possible -- no later than 48 hours after receiving the formal acceptance. Your manuscript will remain under strict press embargo until 2 pm Eastern Time on the date of publication. For more information, please contact onepress@plos.org.

Kind regards,

Baeckkyoung Sung, Ph.D.

Academic Editor

PLOS One

Additional Editor Comments (optional):

The revised manuscript has reflected all the requirements suggested by the reviewers.

Reviewers' comments:

Reviewer's Responses to Questions

**Comments to the Author**

1. If the authors have adequately addressed your comments raised in a previous round of review and you feel that this manuscript is now acceptable for publication, you may indicate that here to bypass the “Comments to the Author” section, enter your conflict of interest statement in the “Confidential to Editor” section, and submit your "Accept" recommendation.

Reviewer #2: (No Response)

**********

2. Is the manuscript technically sound, and do the data support the conclusions?

The manuscript must describe a technically sound piece of scientific research with data that supports the conclusions. Experiments must have been conducted rigorously, with appropriate controls, replication, and sample sizes. The conclusions must be drawn appropriately based on the data presented.

Reviewer #2: (No Response)

**********

3. Has the statistical analysis been performed appropriately and rigorously?

Reviewer #2: (No Response)

**********

4. Have the authors made all data underlying the findings in their manuscript fully available?

The PLOS Data policy requires authors to make all data underlying the findings described in their manuscript fully available without restriction, with rare exception (please refer to the Data Availability Statement in the manuscript PDF file). The data should be provided as part of the manuscript or its supporting information, or deposited to a public repository. For example, in addition to summary statistics, the data points behind means, medians and variance measures should be available. If there are restrictions on publicly sharing data—e.g. participant privacy or use of data from a third party—those must be specified.

Reviewer #2: (No Response)

**********

5. Is the manuscript presented in an intelligible fashion and written in standard English?

PLOS ONE does not copyedit accepted manuscripts, so the language in submitted articles must be clear, correct, and unambiguous. Any typographical or grammatical errors should be corrected at revision, so please note any specific errors here.

Reviewer #2: (No Response)

**********

6. Review Comments to the Author

Please use the space provided to explain your answers to the questions above. You may also include additional comments for the author, including concerns about dual publication, research ethics, or publication ethics. (Please upload your review as an attachment if it exceeds 20,000 characters)

Reviewer #2: The authors addressed all of my previous comments. I have no more concerns and recommend publication.

**********

7. PLOS authors have the option to publish the peer review history of their article (what does this mean?). If published, this will include your full peer review and any attached files.

If you choose “no”, your identity will remain anonymous but your review may still be made public.

**Do you want your identity to be public for this peer review?**  For information about this choice, including consent withdrawal, please see our Privacy Policy.

Reviewer #2: No

**********

# Editor report: Acceptance letter

**DOI:** 10.1371/journal.pone.0351138.r006

**Authors:** Baeckkyoung Sung

PONE-D-25-58465R2

PLOS One

Dear Dr. Tai,

I'm pleased to inform you that your manuscript has been deemed suitable for publication in PLOS One. Congratulations! Your manuscript is now being handed over to our production team.

At this stage, our production department will prepare your paper for publication. This includes ensuring the following:

* All references, tables, and figures are properly cited

* All relevant supporting information is included in the manuscript submission,

* There are no issues that prevent the paper from being properly typeset

You will receive further instructions from the production team, including instructions on how to review your proof when it is ready. Please keep in mind that we are working through a large volume of accepted articles, so please give us a few days to review your paper and let you know the next and final steps.

Lastly, if your institution or institutions have a press office, please let them know about your upcoming paper now to help maximize its impact. If they'll be preparing press materials, please inform our press team within the next 48 hours. Your manuscript will remain under strict press embargo until 2 pm Eastern Time on the date of publication. For more information, please contact onepress@plos.org.

You will receive an invoice from PLOS for your publication fee after your manuscript has reached the completed accept phase. If you receive an email requesting payment before acceptance or for any other service, this may be a phishing scheme. Learn how to identify phishing emails and protect your accounts at https://explore.plos.org/phishing.

If we can help with anything else, please email us at customercare@plos.org.

Thank you for submitting your work to PLOS ONE and supporting open access.

Kind regards,

PLOS ONE Editorial Office Staff

on behalf of

Dr. Baeckkyoung Sung

Academic Editor

PLOS One
